# Benefits of targeted deployment of physician- led interprofessional pre-hospital teams on the care of critically ill and injured patients: a systematic review and meta-analysis - matters arising response

**DOI:** 10.1186/s13049-025-01355-w

**Published:** 2025-03-17

**Authors:** Matthew D. Lavery, Arshbir Aulakh, Michael D. Christian

**Affiliations:** University of British Columbia, Vancouver, Canada

Dear Editor,

Thank you for the opportunity to respond to the comments on our recent paper published by McHenry [1] and Boulton et al. [2], respectively. We are grateful they have taken the time to share their feedback and are encouraged that their findings both align with and further support those we published.

Both McHenry [1] and Boulton et al. [2] have rightly pointed out the slight awkwardness of how we presented our findings in two individual groups based on whether the original research analysed their findings regarding mortality or survival. McHenry has very effectively independently analysed the studies and found an overall 41% improved survival with the targeted deployment of physician-led interprofessional prehospital critical care teams [OR 1.41, 95%CI 1.27–1.56]. In hindsight, we agree that this approach to the analysis provides further clarity. However, in conducting our analysis, we felt it was important to take the more conservative approach so as not to further extend the methodological or statistical limits of the original research as well as to allow the readers to more directly compare the inputs we used in our analysis to the original publications. Ultimately however, we agree that McHenry’s approach is a valuable addition to the literature.

Boulton et al. [2] additionally commented, referencing their recent excellent publication of a meta-analysis of the impact of prehospital critical care on the outcomes of out-of-hospital-cardiac-arrest (OHCA), which was found to improve 30-day survival by 56% (OR 1.56, 95% CI 1.38–1.75) [3]. They highlighted that fifteen of the sixteen studies included in their meta-analysis included physician-led interprofessional critical care teams. However, our analysis did not include nine of these studies. [4–12] Although similar, the focus of their question and associated inclusion and exclusion criteria differs significantly from our study. We have reviewed our analysis and found that six [4–8, 12] of the studies were identified in our search but excluded due to their methods or outcome measures (see Table 1– online supplement). Of the three papers we failed to identify in our search, one [9] does not compare the models of care directly and thus would have been excluded from our analysis. The other two papers [10, 11] meet our includes and exclusion criteria and should have been included in our analysis. We believe these were missed by the search engines, likely due to the terminology used in their titles and keywords. Both papers reported point estimates indicating a survival benefit for physician-led interprofessional critical care teams, ORs 1.06 and 1.54 for survival to hospital discharge, but were said to be not ‘statistically significant’, although this is most likely due to being underpowered.

Boulton et al. [3] attributed the improved outcomes to the pre-hospital application of critical care treatments. However, since 93.8% of the papers in their study involved physician-led interprofessional teams, we question whether they may have overlooked or underestimated the impact of interprofessional teams [13] in the discussion of their findings.

Sincerely,

Matthew Lavery, Arshbir Aulakh, Michael D. Christian.

## Electronic supplementary material

Below is the link to the electronic supplementary material.

Supplementary Material 1

## Data Availability

No datasets were generated or analysed during the current study.
