# Supplementary material for: Benefits of targeted deployment of physician- led interprofessional pre-hospital teams on the care of critically ill and injured patients: a systematic review and meta-analysis - matters arising response
Source: Scand J Trauma Resusc Emerg Med. 2025 Mar 17;33:46. doi: 10.1186/s13049-025-01355-w (PMC11916955; doi:10.1186/s13049-025-01355-w)
Supplement: Supplementary file 1 — Supplementary Material 1 [file 13049_2025_1355_MOESM1_ESM.docx]

On-line supplemental material

Table 1.

| **Did we find the article?** | **Title** | **Citation** | **Reason for exclusion or failure to find article** |
| --- | --- | --- | --- |
| Yes | Prehospital determinants of successful resuscitation after traumatic and non-traumatic out-of-hospital cardiac arrest. | Barnard EB, Sandbach DD, Nicholls TL, Wilson AW, Ercole A. Prehospital determinants of successful resuscitation after traumatic and non-traumatic out-of-hospital cardiac arrest. Emergency Medicine Journal. 2019 Jun 1;36(6):333-9. | Exclude: It focuses on understanding the determinants of survival in out-of-hospital cardiac arrest (OHCA) and the impact of interventions like bystander CPR and air ambulance services. It does not directly address the comparison between physician-manned immediate care teams and the current system. |
| Yes | Effect of replacing ambulance physicians with paramedics on outcome of resuscitation for prehospital cardiac arrest | Bjornsson HM, Bjornsdottir GG, Olafsdottir H, Mogensen BA, Mogensen B, Thorgeirsson G. Effect of replacing ambulance physicians with paramedics on outcome of resuscitation for prehospital cardiac arrest. European Journal of Emergency Medicine. 2021 Jun 1;28(3):227-32. | Outcome not listed as AOR. Further the physician critical care arm took place nine years prior to publication and well before the 2010 inclusion date. |
| Yes | Epidemiology, management and survival outcomes of adult out-of-hospital traumatic cardiac arrest due to blunt, penetrating or burn injury | Doan TN, Wilson D, Rashford S, Sims L, Bosley E. Epidemiology, management and survival outcomes of adult out-of-hospital traumatic cardiac arrest due to blunt, penetrating or burn injury. Emergency medicine journal. 2022 Feb 1;39(2):111-7. | Comparison of CCP to high acuity CCP (with occasional physician presence). |
| Yes | Neurological outcomes in patients transported to hospital without a prehospital return of spontaneous circulation after cardiac arrest | Goto Y, Maeda T, Nakatsu-Goto Y. Neurological outcomes in patients transported to hospital without a prehospital return of spontaneous circulation after cardiac arrest. Critical Care. 2013 Dec;17:1-10. | Published prior to 2015. Also, this isn't primarily a comparison of the model of care but rather just one variable that is only applicable to a small number of patients. |
| Yes | Prehospital Physician Presence for Patients With out-of-Hospital Cardiac Arrest Undergoing Extracorporeal Cardiopulmonary Resuscitation: A Multicenter, Retrospective, Nationwide Observational Study in Japan (The JAAM–OHCA registry) | Nakajima S, Matsuyama T, Watanabe M, Komukai S, Kandori K, Okada A, Okada Y, Kitamura T, Ohta B. Prehospital Physician Presence for Patients With out-of-Hospital Cardiac Arrest Undergoing Extracorporeal Cardiopulmonary Resuscitation: A Multicenter, Retrospective, Nationwide Observational Study in Japan (The JAAM–OHCA registry). Current Problems in Cardiology. 2023 May 1;48(5):101600. | Excluded as the study included a nieche cohort of patients who underwent ECMO and as a resukt, it would be very heterogeneous from the other studies. |
| No | Pre-hospital predictors of long-term survival from out-of-hospital cardiac arrest | Pemberton K, Franklin RC, Bosley E, Watt K. Pre-hospital predictors of long-term survival from out-of-hospital cardiac arrest. Australasian Emergency Care. 2023 Jun 1;26(2):184-92. | Does not compare physician interdisciplinary teams to paramedic models of care. |
| No | The impact of a pre-hospital critical care team on survival from out-of-hospital cardiac arrest | von Vopelius-Feldt J, Coulter A, Benger J. The impact of a pre-hospital critical care team on survival from out-of-hospital cardiac arrest. Resuscitation. 2015 Nov 1;96:290-5. | “Critical Care Team” referenced in the abstract instead of physician staffed etc so was not caught by our search terms. “Physician” not included in key words. |
| No | The effect of prehospital critical care on survival following out-of-hospital cardiac arrest: A prospective observational study | von Vopelius-Feldt J, Morris RW, Benger J. The effect of prehospital critical care on survival following out-of-hospital cardiac arrest: A prospective observational study. Resuscitation. 2020 Jan 1;146:178-87. | “Critical Care Team” referenced in the abstract instead of physician staffed etc so was not caught by our search terms. “Physician” not included in key words. |
| Yes | Collaborative effects of bystander-initiated cardiopulmonary resuscitation and prehospital advanced cardiac life support by physicians on survival of out-of-hospital cardiac arrest: a nationwide population-based observational study | Yasunaga H, Horiguchi H, Tanabe S, Akahane M, Ogawa T, Koike S, Imamura T. Collaborative effects of bystander-initiated cardiopulmonary resuscitation and prehospital advanced cardiac life support by physicians on survival of out-of-hospital cardiac arrest: a nationwide population-based observational study. Critical Care. 2010 Dec;14:1-8. | In this study, there is a confounding variable (with or without BCPR), and no direct comparison betwen Group A and B is not statistically significant. |
